# Early Post-stroke Cognition: In-hospital Predictors and the Association With Functional Outcome

**DOI:** 10.3389/fneur.2020.613607

**Published:** 2020-12-23

**Authors:** Richa Sharma, Dania Mallick, Rafael H. Llinas, Elisabeth B. Marsh

**Affiliations:** ^1^Department of Neurology, Yale University School of Medicine, New Haven, CT, United States; ^2^Department of Neurology, The Johns Hopkins School of Medicine, Baltimore, MD, United States

**Keywords:** stroke, recovery, cognition, rehabilitation, outcomes

## Abstract

**Purpose:** To characterize and predict early post-stroke cognitive impairment by describing cognitive changes in stroke patients 4–8 weeks post-infarct, determining the relationship between cognitive ability and functional status at this early time point, and identifying the in-hospital risk factors associated with early dysfunction.

**Materials and Methods:** Data were collected for 214 patients with ischemic stroke and 39 non-stroke controls. Montreal Cognitive Assessment (MoCA) exams were administered at post-hospitalization clinic visits approximately 4–8 weeks after infarct. MoCA scores were compared for patients with: no stroke, minor stroke [NIH Stroke Scale (NIHSS) < 5], and major stroke. Ordinal logistic regression was performed to assess the relationship between MoCA score and functional status [modified Rankin Scale score (mRS)] at follow-up. Predictors of MoCA < 26 and < 19 (cutoffs for mild and severe cognitive impairment, respectively) at follow-up were identified by multivariable logistic regression using variables available during hospitalization.

**Results:** Post stroke cognitive impairment was common, with 66.8% of patients scoring < 26 on the MoCA and 22.9% < 19. The average total MoCA score at follow-up was 18.7 (SD 7.0) among major strokes, 23.6 (SD 4.8) among minor strokes, and 27.2 (SD 13.0) among non-strokes (*p* = <0.0001). The follow-up MoCA score was associated with the follow-up mRS in adjusted analysis (OR 0.69; 95% C.I. 0.59–0.82). Among patients with no prior cognitive impairment (*N* = 201), a lack of pre-stroke employment, admission NIHSS > 6, and left-sided infarct predicted a follow-up MoCA < 26 (c-statistic 0.75); while admission NIHSS > 6 and infarct volume > 17 cc predicted a MoCA < 19 (c-statistic 0.75) at follow-up.

**Conclusion:** Many patients experience early post-stroke cognitive dysfunction that significantly impacts function during a critical time period for decision-making regarding return to work and future independence. Dysfunction measured at 4–8 weeks can be predicted during the inpatient hospitalization. These high-risk individuals should be identified for targeted rehabilitation and counseling to improve longer-term post-stroke outcomes.

## Introduction

There is a known link between ischemic stroke and long-term cognitive impairment. The prevalence of dementia in patients with a history of ischemic stroke is nearly thirty percent; 3.5–5.8 times higher than in patients without a stroke history (1). One follow-up study reported that over a quarter of stroke patients display delayed dementia, while additional studies using more formalized neuropsychological assessments such as the Montreal Cognitive Assessment (MoCA) or Mini-Mental Status Examination have reported rates of cognitive impairment ranging from 35 to 92% (2–4). Impairment has been reported to affect multiple cognitive domains (5), though a recent study evaluating those with transient ischemic attack and minor stroke found difficulty with executive function and psychomotor processing to be the most common cognitive deficits (6). Cognitive dysfunction in stroke patients can result in deficits such as impaired activities of daily living (ADLs) and instrumental activities of daily living (IADLs), lost wages, increased health care costs, loss of independence, and social isolation.

A recent retrospective study showed an association between MoCA scores documented acutely after stroke and functional outcome during rehabilitation (6). Our study takes the logical next step and addresses the ability of in-hospital variables to predict cognitive impairment and functional status once patients have been discharged home from the inpatient setting and are transitioning back to a “normal life.” These early months represent a critical time when patients and families make decisions regarding returning to work, living alone, and functioning independently. Prior studies have identified numerous factors associated with long-term cognitive impairment in stroke patients including: white matter disease (7), lower educational level, older age, female sex, recurrent stroke, and global cortical atrophy in patients with ischemic stroke and vascular cognitive impairment (8). But, the ability to predict cognitive dysfunction that may impact recovery, at the time of hospitalization, would potentially enable physicians to identify those at risk for early cognitive decline, before life-altering decisions are made impacting quality of life.

This study aims to: (1) assess cognition across multiple domains during the early phase of stroke recovery (first 1–6 months) among patients with major strokes, minor strokes, and non-stroke controls using a simple and efficient screen (the MoCA), (2) confirm the MoCA score at early follow-up is associated with concurrent functional status (modified Rankin Scale score), and (3) identify risk factors available during the stroke hospitalization predictive of early cognitive impairment at follow-up among those without a prior history of cognitive impairment.

## Materials and Methods

This study was approved by our Institutional Review Board and was performed in accordance with STROBE criteria. Given the observational nature of the study and that data were collected as part of routine clinical care and stored in a HIPAA-approved clinical research database, informed consent was not required. Any reasonable request for data sharing can be directed to the corresponding author.

### Study Population

A prospectively-collected series of patients presented to the Johns Hopkins Bayview Stroke Intervention Clinic (BASIC) in Baltimore, Maryland from January 2013 to August 2018 between 1 and 6 months after hospitalization for a symptomatic, acute ischemic stroke that was radiographically evident on noncontrast head computed tomography (CT) or magnetic resonance imaging (MRI). BASIC is a multidisciplinary stroke follow-up clinic that typically sees patients 4–8 weeks post-stroke. Patients are evaluated by a board-certified vascular neurologist and rehabilitation specialists (as needed). They undergo formal neurological evaluation along with assessment of mood, fatigue, cognition, and functional ability. Individuals were excluded from the study cohort if they were unable to participate in cognitive testing due to global aphasia, severe visual impairment, decreased level of alertness, or had intracranial hemorrhage on neuroimaging (CT or MRI). The MoCA patterns of these ischemic stroke patients were compared with a cohort of non-stroke patients with no prior history of neurodegenerative or psychiatric illness who presented to stroke clinic with diagnoses of transient ischemic attack, seizure, or migraine.

### Defining Cognitive Impairment

At their follow-up clinic visit, all patients underwent a basic neurological examination and were administered the MoCA, the validity of which has been demonstrated in stroke patients (9, 10). The total MoCA score along with scores for each domain of cognition (visuospatial/executive, naming, attention, language, abstraction, recall, and orientation) were recorded for each patient. “Any cognitive impairment” was defined as total a MoCA score of <26 (11). “Severe impairment” was defined as total MoCA scores of <19 (12). The MoCA was administered by trained clinic personnel who were blinded to the patient's modified Rankin Scale score during the clinic visit.

### Covariates

Demographic, clinical, and radiologic covariates were collected including: demographics (age, sex, race, marital status, education, pre-stroke employment status, household income approximated by median income of the patient's zip code); stroke characteristics (severity- admission National Institute of Health Stroke Scale (NIHSS) score, functional status- modified Rankin scale (mRS) score at baseline, stroke volume, lesion location, degree of chronic white matter disease, presence of cerebral microbleeds); and medical history [prior stroke, pre-existing history of cognitive impairment or dementia, vascular comorbidities, Charlson Comorbidity Index (13), anti-depressant use]. Modified Rankin Scale score was also documented at the stroke clinic follow-up appointment by the patient's neurologist who was blinded to the patient's clinic MoCA result.

### Neuroimaging

Masked review of available CT and MRI was performed to calculate stroke volume and other radiographic factors by a board-certified vascular neurologist blinded to patient outcomes. The neuroimaging was obtained during hospital admission. The volume of a hypodensity on CT or diffusion restriction on MRI matching the vascular distribution of the clinical stroke was calculated by the ellipsoid ABC/2 method (14). On MRI, the T2 FLAIR sequence was also utilized to calculate the Cardiovascular Health Study (CHS) score to estimate the degree of white matter disease burden or leukoaraiosis using the CHS study library of templates (15). Studies were graded from 1 (barely detectable white matter changes) to 8 (extensive, confluent changes). Any evidence of large vessel infarct or lacunar infarct was excluded from this classification (16, 17). The presence of cerebral microhemorrhages by hemosiderin deposits were identified on gradient echo sequences as lesions ≤10 mm in diameter.

### Statistical Analysis

All statistical analyses were performed using SAS 9.4 (Cary, NC). Analysis of variance (ANOVA) testing was performed to compare total MoCA scores and domain-specific scores among patients with: no ischemic infarct, minor stroke (admission NIHSS ≤4), and major stroke (admission NIHSS >4). Additional comparisons across groups were performed using ANOVA testing for continuous variables and Pearson's Chi-square tests for categorical variables.

To assess the association between the follow-up total MoCA score obtained in clinic (the independent predictor of interest) as well as other factors, and functional status at that timepoint, we performed univariable and multivariable ordinal logistic regression analyses given the non-normal distribution of the variable, mRS. Variables significant in univariable analysis (*p* < 0.05) were included in subsequent multivariable logistic regression analysis along with the MoCA score to adjust for potential confounders that may contribute to functional status at follow-up after stroke.

Further analyses were undertaken for the subgroup of patients with ischemic stroke and no prior history of cognitive impairment. Univariable logistic regressions were performed to predict two outcome measures: total MoCA score < 26 and MoCA score < 19. We then created multivariable logistic regression models using variables significant in univariable analyses for each outcome if the *p*-value in univariable analysis was < 0.2. To prevent overfitting, variables were selected to be included in the final models by stepwise selection.

## Results

### Characteristics of Ischemic Stroke Patients and Non-stroke Controls

Over the recruitment period, 253 patients presented to follow-up clinic and were eligible for enrollment: 214 ischemic stroke patients and 39 non-stroke controls. Differences between patients with major and minor stroke and non-stroke controls are displayed in Table 1. The non-stroke controls consisted of patients with the following diagnoses: migraine (*N* = 16); transient ischemic attack (*N* = 10); seizure (*N* = 7); and post-concussive headache (*N* = 1). Patients within the stroke cohort were significantly older, were less educated, had lower household incomes, had higher baseline mRS scores, and had more vascular risk factors compared to the non-stroke cohort, but had similar degrees of white matter disease compared to controls. Those with major strokes were less likely to be employed at baseline than those with minor stroke or mimics. The mean time from symptom onset to clinic visit was 1.3 months for those with minor strokes and 1.2 months for patients with major strokes. Although stroke patients were only included if time from stroke to follow-up was ≤6 months, >80% of stroke patients were evaluated within 90 days of symptom onset. The mean volume of infarct was significantly higher among those with major (31.7 cc) vs. minor (9.8 cc) stroke, and patients with greater stroke severity were more likely to have suffered a cortical infarct. There was no difference in leukoaraiosis or microbleed burden among stroke patients by stroke severity.

**Table 1:** Baseline demographic, clinical, and radiographic characteristics (*N* = 253).

**Variable**	**Minor infarct (*N* = 141)**	**Major infarct (*N* = 73)**	**No infarct (*N* = 39)**	***P*-value**
**Demographics**				
Age (mean years, SD)	63.2 (14.2)	64.7 (13.8)	57.6 (18.2)	0.047
Male sex (*N*, %)	59 (41.8)	40 (54.8)	16 (41.0)	0.292
Caucasian race (*N*, %)	112 (79.4)	33 (84.6)	33 (84.6)	0.144
Education >12th grade (N, %)	72 (55.4)	32 (52.5)	34 (91.9)	<0.001
Household median income (mean, SD)	55,130.3 (18,630.7)	52,002.6 (17,930.8)	74,485.0 (30,225.6)	<0.001
Employed (*N*, %)	52 (38.8)	12 (17.1)	14 (35.9)	0.006
Baseline mRS (mean)	1.2 (1.20)	2.3 (1.7)	0.8 (1.1)	<0.001
Lives alone (*N*, %)	23 (16.4)	16 (21.9)	12 (30.8)	0.131
Time to follow-up (mean months, SD)	1.3 (4.9)	1.2 (2.4)	–	0.997
**Comorbidities**				
Diabetes (N, %)	59 (41.8)	25 (34.3)	6 (15.4)	0.009
Hypertension (*N*, %)	109 (77.3)	59 (80.8)	19 (48.7)	<0.001
Hyperlipidemia (*N*, %)	112 (79.4)	52 (71.2)	22 (56.4)	0.014
Active smoker (*N*, %)	51 (36.2)	35 (48.0)	13 (33.3)	0.178
Prior stroke (*N*, %)	53(37.9)	33 (45.2)	8 (20.5)	0.036
Prior dementia (*N*, %)	6 (4.3)	7 (9.6)	2 (5.1)	0.286
Current use of anti-depressant (*N*, %)	45 (31.9)	34 (46.6)	19 (48.7)	0.043
Charlson Comorbidity Index, mean (SD)	3.4 (2.6)	4.2 (2.6)	1.8 (2.5)	<0.001
**Clinical metrics**				
Admission NIHSS (mean, SD)	1.8 (1.3)	9.2 (4.8)	–	<0.001
Follow-up NIHSS (mean, SD)	1.3 (2.3)	2.7 (3.5)	–	<0.001
Follow-up mRS (mean, SD)	1.5 (1.3)	2.2 (1.4)	–	<0.001
Length of stay in days (mean, SD)	4.0 (4.3)	7.6 (6.9)	–	<0.001
**Radiographic characteristics**				
Stroke volume (mean cc, SD)	9.8 (21.1)	31.7 (54.6)	–	<0.001
Laterality, left (*N*, %)	63 (48.8)	30 (42.3)	–	0.372
Cortical infarct (*N*, %)	72 (51.4)	52 (71.2)	–	0.005
CHS score (mean, SD)	3.0 (1.8)	3.3 (2.0)	2.7 (1.8)	0.379
Microbleeds present (*N*, %)	21 (23.9)	16 (36.4)	2 (12.5)	0.127

*mRS, modified Rankin score; NIHSS, National Institutes of Health Stroke Severity; CMB, cerebral microbleed; CHS, Cardiovascular Health Study*.

**Figure 1:**
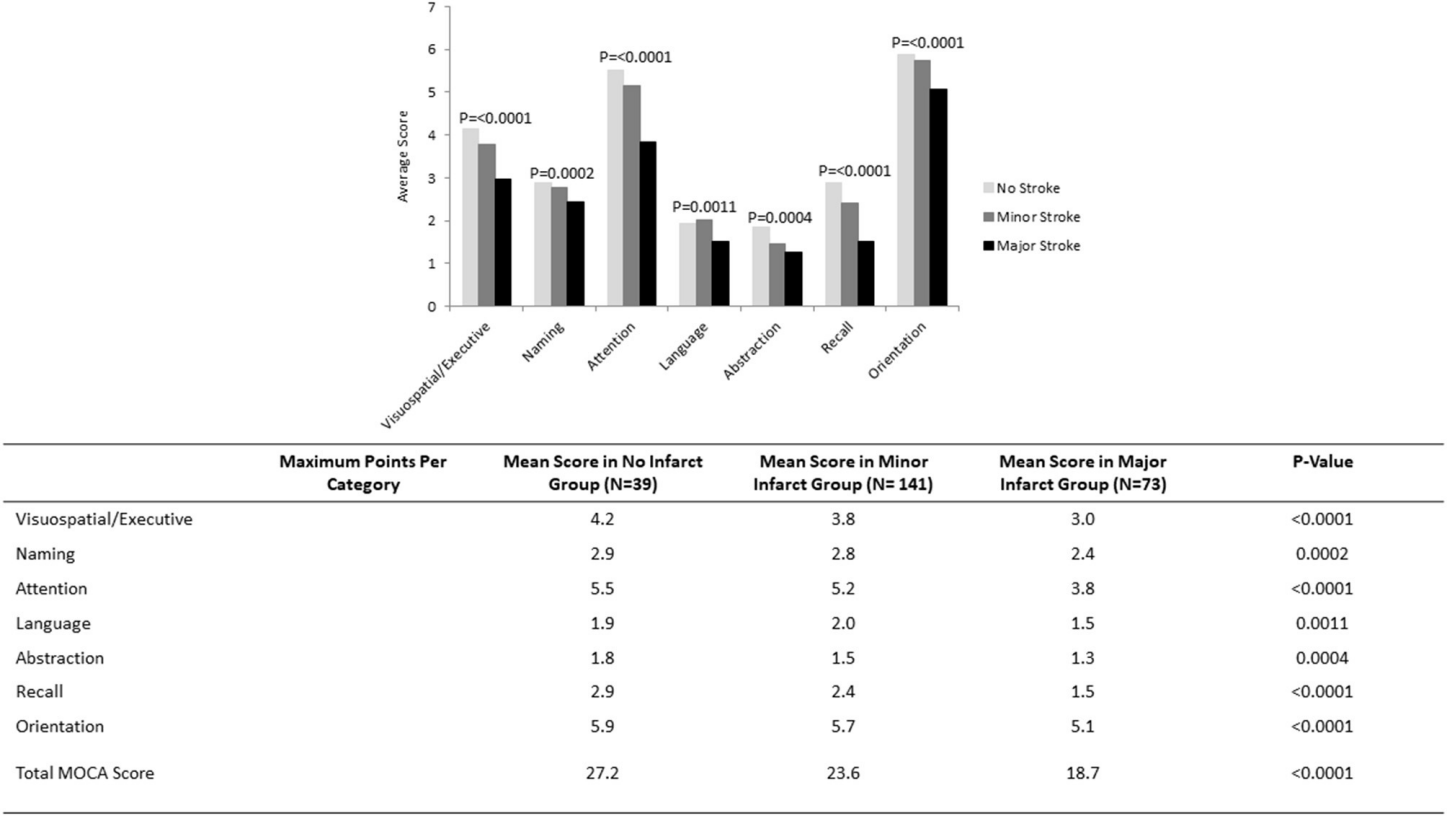
MoCA scores by cognitive domain in subgroups of patients with no infarct (*N* = 39), minor stroke (*N* = 141), and major stroke (*N* = 73).

Nearly two-thirds (*n* = 143, 66.8%) of patients with stroke had a MoCA score of <26 at follow-up; while 49 (22.9%) had a MoCA score of <19. Numbers were highest in those with major stroke (*n* = 60, 82.2% for MoCA < 26; *n* = 31, 42.5% for MoCA < 19) compared to minor stroke (*n* = 83, 58.9% for MoCA < 26; *n* = 18, 12.8% for MoCA < 19). Cognitive impairment was also present in a portion of controls, but less common and less severe (*n* = 20, 51.3% MoCA < 26; *n* = 2, 5.1% MoCA < 19). The average total MoCA score was significantly lower for patients with major (mean 18.7, SD 7.0) and minor (mean 23.6, SD 4.8) strokes compared to non-stroke controls (mean 27.2, SD 13.0). Patients with no stroke performed significantly better across all cognitive domains with the exception of language compared to patients with minor stroke, who in turn performed better than patients with major stroke (Figure 1).

### Association Between the Follow-Up MoCA and Functional Status at That Timepoint

In univariable ordinal logistic regression analysis of patients with ischemic stroke, the MoCA score in clinic was significantly associated with follow-up mRS score (Table 2, OR 0.83; 95% C.I. 0.80–0.87). When adjusted for possible confounders including NIHSS score, demographic, clinical, and radiographic covariates, the follow-up MoCA score remained an independent and significant predictor of functional status at that follow-up timepoint (OR 0.69; 95% C.I. 0.59–0.82; c-statistic 0.93).

**Table 2:** Unadjusted and adjusted associations between variables and modified Rankin Scale score at follow-up.

**Variable**	**Unadjusted Odds Ratio (95% CI)**	***P*-value**	Adjusted Odds Ratio^*^ (95% CI)	***P*-value**
Follow-up MoCA	0.83 (0.80–0.87)	<0.001	0.69 (0.59–0.82)	<0.001
Follow-up NIHSS	1.29 (1.18–1.41)	<0.001	1.93 (1.40–2.67)	<0.001
Age	1.03 (1.01–1.04)	<0.001	0.95 (0.90–0.99)	0.033
Sex	1.12 (0.72–1.75)	0.623	0.37 (0.10–1.35)	0.133
Education	0.51 (0.31–0.82)	0.006	0.29 (0.09–0.97)	0.045
White race	1.13 (0.66–1.93)	0.660	9.33 (2.05–42.41)	0.004
Median household income < $50,000	2.23 (1.40–3.56)	<0.001	0.85 (0.27–2.68)	0.780
Employment	0.29 (0.17–0.48)	<0.001	0.18 (0.05–0.71)	0.015
Lives alone	0.58 (0.33–1.03)	0.061	3.28 (0.67–16.08)	0.143
Hypertension	5.13 (2.94–8.96)	<0.001	8.04 (1.03–63.04)	0.047
Hyperlipidemia	1.74 (1.04–2.90)	0.035	0.38 (0.05–2.63)	0.324
Diabetes	1.31 (0.82–2.09)	0.255	1.14 (0.37–3.49)	0.817
Smoking	1.68 (1.06–2.66)	0.027	0.64 (0.21–1.99)	0.440
Prior stroke	2.35 (1.47–3.76)	<0.001	1.35 (0.47–3.89)	0.584
Prior dementia	2.75 (1.09–6.97)	0.033	1.05 (0.07–16.31)	0.972
Anti-depressant use	1.31 (0.83–2.08)	0.242	1.25 (0.41–3.79)	0.695
Charlson Comorbidity Index	1.18 (1.03–1.35)	0.015	1.09 (0.84–1.41)	0.539
Baseline mRS	2.19 (1.81–2.65)	<0.001	2.02 (1.25–3.26)	0.004
Infarct volume	1.01 (1.00–1.02)	0.002	1.04 (1.01–1.07)	0.014
Laterality, left	1.38 (0.86–2.21)	0.180	0.91 (0.34–2.48)	0.854
Cortical	0.91 (0.56–1.48)	0.712	0.11 (0.03–0.39)	<0.001
CHS score	1.18 (1.03–1.35)	0.015	0.83 (0.60–1.15)	0.261
CMB count	1.25 (1.08–1.45)	0.003	1.37 (1.10-1.70)	0.004

*CI, confidence interval; MoCA, Montreal Cognitive Assessment; NIHSS, National Institutes of Health Stroke Severity; mRS, modified Rankin score; CHS, Cardiovascular Health Study; CMB, cerebral microbleed*.

* *Adjusted for factors significant in univariable analysis (p < 0.05): age, sex, race, education, household income, employment status prior to stroke, marital status, living alone, medical comorbidities, Charlson Comorbidity Index, follow-up NIHSS, baseline mRS, stroke volume, laterality of stroke, cortical location of stroke, CHS score, and CMB count*.

### Predictors of Follow-Up MoCA Scores

There were 201 ischemic stroke patients with no prior history of cognitive impairment in our cohort. Of these patients, 132 scored <26 (any cognitive impairment) and 41 scored <19 (severe impairment) on follow-up MoCA examination. Univariable regression analyses of factors predictive of a new diagnosis of mild cognitive impairment or MCI and severe impairment are displayed in Table 3. Continuous variables significant in univariable analyses were then dichotomized using their sample mean value (age > 65, baseline mRS > 2, Charlson Comorbidity Index > 3, stroke volume > 17 cc, CHS score > 2, microbleed > 0) with the exception of admission NIHSS, where a cut point of 6 was chosen based on previously published work (18). Multivariable logistic regression modeling with stepwise selection using the variables significant in univariable analyses yielded 2 separate models predicting MoCA < 26 and MoCA < 19 (Table 4). Employment prior to stroke (OR 0.28; 95% C.I. 0.12–0.69), admission NIHSS > 6 (OR 4.48; 95% C.I. 1.15–17.47), and left-sided location of infarct (OR 2.85; 95% C.I. 1.17–6.95) were significant and independent predictors of MoCA < 26 with a model c-statistic of 0.75. MoCA < 19 was predicted by admission NIHSS > 6 (OR 7.00; 95% C.I. 2.53–19.40) and ischemic stroke volume > 17 cc (OR 3.60; 95% C.I. 1.24–10.47) with a model c-statistic of 0.75.

**Table 3:** Univariable logistic regression analyses of the odds of at least mild cognitive impairment (MoCA < 26) and the odds of severe cognitive impairment (MoCA < 19) associated with each covariate in patients after ischemic stroke with no prior diagnosis of cognitive impairment (*N* = 201).

**Variable**	**Unadjusted odds ratio of any cognitive impairment (*N* = 132)**	**95% CI**	***P*-value**	**Unadjusted odds ratio of severe cognitive impairment (*N* = 41)**	**95% CI**	***P*-value**
Age	1.04	1.01, 1.06	0.002	1.05	1.02, 1.08	<0.001
Sex	1.05	0.59, 1.89	0.862	1.16	0.59, 2.31	0.665
Caucasian race	0.58	0.28, 1.21	0.150	0.58	0.27, 1.24	0.161
Education	0.51	0.28, 0.98	0.043	0.77	0.36, 1.63	0.491
Median household income < $50,000	1.26	0.68, 2.33	0.459	3.50	1.39, 8.81	0.008
Employment	0.51	0.27, 0.95	0.033	0.48	0.21, 1.12	0.091
Marital status	1.73	0.60, 5.02	0.313	0.67	0.20, 2.24	0.520
Living alone	0.67	0.32, 1.40	0.285	0.74	0.28, 1.91	0.531
Diabetes	1.04	0.58, 1.89	0.889	0.65	0.31, 1.34	0.238
Hypertension	1.93	0.97, 3.85	0.060	2.98	1.00, 8.89	0.050
Hyperlipidemia	0.91	0.45, 1.82	0.780	0.56	0.26, 1.20	0.135
Smoking	1.78	0.97, 3.28	0.065	1.93	0.97, 3.86	0.063
Prior stroke	2.21	1.20, 4.09	0.011	1.26	0.63, 2.52	0.518
Anti-depressant use	1.54	0.82, 2.89	0.176	1.22	0.60, 2.48	0.579
Charlson Comorbidity Index	1.25	1.09, 1.43	0.002	1.19	1.06, 1.35	0.004
Admission NIHSS	1.16	1.05, 1.27	0.003	1.18	1.09, 1.27	<0.001
Baseline mRS	1.54	1.20, 1.97	<0.001	1.66	1.26, 2.18	<0.001
Stroke volume	1.02	1.00, 1.03	0.028	1.01	1.00, 1.02	0.034
Laterality, left	2.08	1.12, 3.86	0.021	2.54	1.23, 5.24	0.012
Cortical	1.62	0.90, 2.91	0.110	2.84	1.30, 6.18	0.009
CHS score	1.42	1.15, 1.76	0.001	1.31	1.08, 1.58	0.006
CMB count	4.02	1.34, 12.03	0.013	1.12	0.97, 1.30	0.138

*CI, confidence interval; NIHSS, National Institutes of Health Stroke Severity; mRS, modified Rankin score; CMB, cerebral microbleed; CHS, Cardiovascular Health Study*.

**Table 4:** Multivariable logistic regression models predicting MoCA < 26 *(left)* and MoCA < 19 *(right)* in patients after ischemic stroke without a diagnosis of cognitive impairment prior to the time of ischemic stroke presentation (*N* = 201).

**Predictors**	**MoCA < 26 (*****N*** **= 132)**		**MoCA < 19 (*****N*** **= 41)**	
	**Odds Ratio (95% CI)**	***P*-value**	**Odds Ratio (95% CI)**	***P*-value**
Employment prior to stroke	0.28 (0.12, 0.69)	0.005	–	–
Admission NIHSS scale > 6	4.48 (1.15, 17.47)	0.031	7.00 (2.53, 19.4)	<0.001
Stroke volume > 17 cc	–	–	3.60 (1.24, 10.47)	0.019
Laterality of stroke (left)	2.85 (1.17, 6.95)	0.021	**–**	–

*CI, confidence interval; NIHSS, National Institutes of Health Stroke Severity score*.

*The c-statistic for predicting both MoCA < 26 and < 19 was 0.75*.

## Discussion

This study characterizes impairment across multiple cognitive domains during the early phase of post-stroke recovery. In addition, it proves the logical assumption that cognitive status at follow-up is a significant, independent predictor for functional status at this timepoint. Finally, it identifies risk factors available during hospitalization that predict downstream cognitive dysfunction during the early phase of stroke recovery.

In our study, both the average and domain-specific MoCA scores were significantly lower in ischemic stroke patients vs. non-stroke controls. This finding suggests that the presence of any infarct is associated with at least some degree of early cognitive dysfunction. Nearly two-thirds of ischemic stroke patients with no prior history of cognitive impairment scored below normal on the MoCA (<26). While it is possible that some may have had subclinical dysfunction at baseline, this number is much higher than would have been expected, especially when considering that the average age of the cohort was <65 years. These results are particularly important given the significant relationship between MoCA scores and functional status at the post-stroke clinic visit, even after adjusting for multiple factors including NIHSS scale, emphasizing the impact of cognitive impairment on one's ability to remain independent after stroke. Though early, at 1–2 months post-infarct many patients and families are making decisions that impact the rest of their lives such as living alone or returning to work. Being able to identify those at risk to experience early cognitive dysfunction will allow for targeted rehabilitation focused on cognitive improvement and allow for counseling regarding potential cognitive issues that may or may not resolve over time.

Additional variables improved our ability to predict the degree of cognitive dysfunction post-stroke. The likelihood of any cognitive impairment was predicted by employment status prior to stroke. This is probably a reflection of the individual's premorbid baseline. NIHSS scale at ischemic stroke presentation, infarct volume, and laterality of stroke were also important predictors, likely due to their measure of stroke severity.

Early post-stroke cognitive decline has been reported in the literature with varying trajectories for recovery (19). One prior study suggested rates of cognitive impairment during the initial months of stroke recovery can be as high as 57–67% (20). While this study did not specifically exclude those with pre-stroke cognitive impairment, a study evaluating patients without cognitive impairment prior to stroke found the prevalence of cognitive impairment remained high at 66.4% 2 months post-infarct (21). Collectively, these studies support our findings that early post-stroke cognitive dysfunction is common (22), illustrating the need for better characterization and prediction. Given that recovery from early dysfunction can be variable, chronic post-stroke dementia is typically not diagnosed until more than 6 months after infarct. However, as life-altering decisions are commonly made well before the 6-month time point, we argue it is important to identify early on those at highest risk.

Early cognitive dysfunction post-stroke is particularly important to identify given its association with overall functional status. Classically, the mRS is considered a predominantly motor measure of function, with significant weight placed on ability to ambulate and perform physical activities of daily living. However, we have shown that cognitive status, in conjunction with NIHSS, persisted as an independent and significant predictor of functionality. Prior studies by Saver and Dong evaluated the relationship between the follow-up NIHSS and follow-up mRS as well as baseline MoCA with follow-up mRS, but not the follow-up MoCA with follow-up mRS (23, 24) The relationship between MoCA and mRS may be partly explained by the degree of cognitive function required to successfully care for oneself. Our findings also highlight the inability of the NIHSS to fully capture the range and degree of disability stroke patients suffer in the outpatient setting and suggest that the MoCA should be an important part of the follow-up clinical assessment.

The degree of morbidity attributable to cognitive impairment after a stroke in the follow-up setting provides a compelling justification for early stratification of risk for cognitive impairment after stroke. It is important to identify patients who may benefit from counseling about recovery prognosis as well as more in-depth early cognitive assessment and potential intervention. Patients and families may benefit from cognitive prognostication at the outset to prepare for how cognitive impairment may affect the ability to perform independently activities of daily living and impact the maintenance of overall health due to difficulty with medication and healthy lifestyle adherence. Although the evidence is currently limited, potential rehabilitation strategies that have therapeutic value include: kinematic analysis, memory enhancing tools, and even pharmacologic therapies.

Our study identified demographic, clinical, and radiographic characteristics associated with lower MoCA scores among those with no known pre-stroke cognitive impairment. The mechanism underlying cognitive impairment in stroke patients is yet to be fully elucidated. It has been hypothesized that a stroke may alter neuronal connectivity to the cortex, leading secondarily to cortical thinning and atrophy via myelin loss or axonal damage (25). In our study, we note that the radiographic size of the infarct was a significant predictor of MoCA < 19 but not MoCA < 26. This suggests a threshold capacity of the neuronal reserve that is able to compensate for injured cortex. It also suggests that mild cognitive impairment after stroke may occur regardless of final infarct volume (26), possibly due in part to other factors impacting cognitive reserve. As an example, severe small vessel disease has been identified as a predictor of post-stroke cognitive impairment in the literature and was significant in our univariable analyses, though not after controlling for infarct. Some studies suggest that patients with white matter disease are subject to neurodegeneration due to inflammation of the small blood vessels, and blood-brain barrier breakdown, resulting in the permeation of cytokines into perivascular space and neuronal tissue (27). Additional infarcts may cause further white matter tract injury; however, the effect of leukoaraiosis can be dampened when adjusting for infarct or other variables related to baseline brain function. Accordingly, the CHANGE score (28) included age, education, and presence of chronic lacunar infarct as significant predictors of MOCA < 22 at 3–6 months after ischemic stroke. While age and education were not significant in our study, it is possible that in our population it was employment status that served a similar role to represent an individual's “pre-stroke baseline.”

Interestingly, there were several factors that have been shown to be important predictors of post-stroke dementia that were not associated with early post-stroke cognitive decline. Depression was not a driving factor of impairment in our study, though has been reported to be an important predictor of post-stroke cognitive dysfunction (3). One possible explanation is that nearly half of our cohort was taking an antidepressant at the time of assessment. Additionally, vascular risk factors such as hypertension and diabetes have been found to significantly increase the risk of cognitive impairment after stroke in the long-term, but these were only statistically significant in our analysis in univariable analysis (29). This may illustrate their importance in brain function and long-term recovery, but the relatively greater importance of the presence of the infarct in the *early* disruption of cognition following stroke.

This study is not without limitations. Our cohort was comprised of patients from a single Comprehensive Stroke Center where participants were predominantly Caucasian. In addition, the MoCA test is considered a brief screening tool for cognitive function and may not be the most sensitive metric to detect dysfunction. However, when compared with neuropsychological batteries in patients with strokes due to small vessel disease, there has been a high degree of accuracy in detecting cognitive impairment (28). In addition, the relative brevity may make it an ideal choice for a busy office setting. It is also important to note that given eligibility was predicated on being able to complete the MoCA, there is a possibility of selection bias. While patients with global aphasia were excluded, those with anterior or posterior aphasias may have language impairments that may confound their ability to perform well on the MoCA. Nevertheless, given the prevalence of aphasia as a presenting stroke symptom, including the performance of patients with incomplete aphasias adds to the generalizability of this study. Finally, we included patients with no history of pre-stroke cognitive impairment. These patients did not have formal neuropsychologic testing prior to the occurrence of ischemic stroke and may have had subclinical cognitive impairment, though this is less likely to have significantly biased results given the relatively young age of our cohort.

Despite these limitations, this study provides evidence that there are in-hospital metrics, readily available for all patients, that can be used to predict cognitive impairment during the early phase of stroke recovery, and that this is important due to the strong association between cognition and overall function. Vulnerable patients may be identified at the time of discharge and provided with the necessary resources such as aggressive rehabilitation to potentially improve longer-term outcomes. Future studies are necessary to validate our model in a larger, independent cohort of patients, and to determine whether early identification and aggressive treatment of at-risk individuals will reduce the rate of post-stroke dementia and improve longer-term post-stroke outcomes.

## Conclusion

Cognitive impairment, ranging from mild to severe, is common in the early months after ischemic stroke, and along with NIHSS contributes significantly to a patient's functional status at follow-up. Employment status prior to stroke, NIHSS at stroke presentation, infarct volume, and stroke laterality are strong predictors of dysfunction and may allow us to better identify and target at risk-individuals in the acute setting for aggressive rehabilitation.

## Data Availability Statement

The raw data supporting the conclusions of this article will be made available by the authors, without undue reservation.

## Ethics Statement

The studies involving human participants were reviewed and approved by Johns Hopkins Hospital Institutional Review Board. Given the observational nature of the study, informed consent was not required.

## Author Contributions

RS performed study design, data collection, data analysis, and manuscript writing. EM performed study design and manuscript writing. RL performed significant manuscript editing. DM performed data collection and manuscript editing. All authors contributed to the article and approved the submitted version.

## Conflict of Interest

The authors declare that the research was conducted in the absence of any commercial or financial relationships that could be construed as a potential conflict of interest.
